# Supplementary material for: Metabolomics Markers of COVID-19 Are Dependent on Collection Wave
Source: Metabolites. 2022 Jul 30;12(8):713. doi: 10.3390/metabo12080713 (PMC9415837; doi:10.3390/metabo12080713)
Supplement: Supplementary file 1 [file metabolites-12-00713-s001.zip › metabolites-1823351-supplementary.pdf]

Targeted metabolomics of COVID-19 patient serum samples – an investigation across collection waves

Holly-May Lewis[1], Yufan Liu[1], Cecile F. Frampas[1], Katie Longman[1], Matt Spick[1], Alexander Stewart[2], Emma Sinclair[2], Nora Kasar[2], Danni Greener[3], Anthony D. Whetton[2], Perdita E. Barran[4], Tao Chen[1], Deborah Dunn-Walters[2], Debra J. Skene[2] and Melanie J. Bailey[1]

[1] Faculty of Engineering and Physical Sciences, University of Surrey, Guildford, GU2 7XH, UK
[2] Faculty of Health and Medical Sciences, University of Surrey, Guildford, GU2 7XH, UK
[3] Frimley Park Hospital, Frimley Health NHS Trust, GU16 7UJ, UK
[4] Manchester Institute of Biotechnology, University of Manchester, M1 7DN, United Kingdom
Corresponding Author: Professor Melanie Bailey, tel +44 (0)1483 682593, m.bailey@surrey.ac.uk, Faculty of Engineering and Physical Sciences, University of Surrey, Guildford, GU2 7XH, UK

Table of contents:

Figure/Table	Description	Page
Supplementary Table S1	A table to show the patients who supplied longitudinal samples and the number of days since the first hospital sample was taken	2
Supplementary Table S2	Search Space for GridSearchCV algorithm	2
Supplementary Figure S1	Principal component analysis (PCA) of all of the data (n=164) colour coded by RT-PCR test result where red data points are positive patients (n=193) and green are patients who tested negative (n=41)	3
Supplementary Figure S2	Orthogonal partial least squares discriminant analysis (OPLS-DA) of all of the data (n=164) colour coded by RT-PCR test result, after reclassifying the three prior positives to positive data points, where red data points are positive patients (n=197) and green are patients who tested negative (n=37)	3
Supplementary Figure S3	Orthogonal partial least squares discriminant analysis (OPLS-DA) of the patients with a positive RT-PCR test colour coded by whether they were treated with dexamethasone (YES – blue) or not (NO – green)	4
Supplementary Figure S4	Box plots to show the 3 metabolites that show no significant difference after recovery for the 7 patients with longitudinal samples where (A) First sample taken in hospital, (B) average of samples over hospital stay, (C) longitudinal samples, (D) all negative patients and (E)) Healthy controls (ns – not significant)	4

Supplementary Table S1: A table to show the patients who supplied longitudinal samples and the number of days since the first hospital sample was taken

Patient identification number	1st sample (day)	Repeat samples in hospital (day)	Longitudinal (day)
222	0	2	158
224	0	2	103
225	0	2	107
247	0	2	68
293	0	2	60
328	0	2	219
398	0	2	107

Supplementary Table S2: Search Space for GridSearchCV algorithm

Search Space				
Algorithm	Hyperparameters			
K-Neighbours Classifier	n_neighbors	3, 4, 5, 6, 7, 8, 9	weights	uniform, distance
Linear SVC	penalty	l1, l2	loss	hinge, squared_hinge
Logistic Regression	penalty	l1, l2, elasticnet	C	0.01, 0.1, 1, 10, 100, 1000, 10000
Random Forest Classifier	n_estimators	100, 1000	max_features	1, 2
Decision Tree Classifier	splitter	best, random	max_depth	3, 6, 9, 12, 15, 18, 21

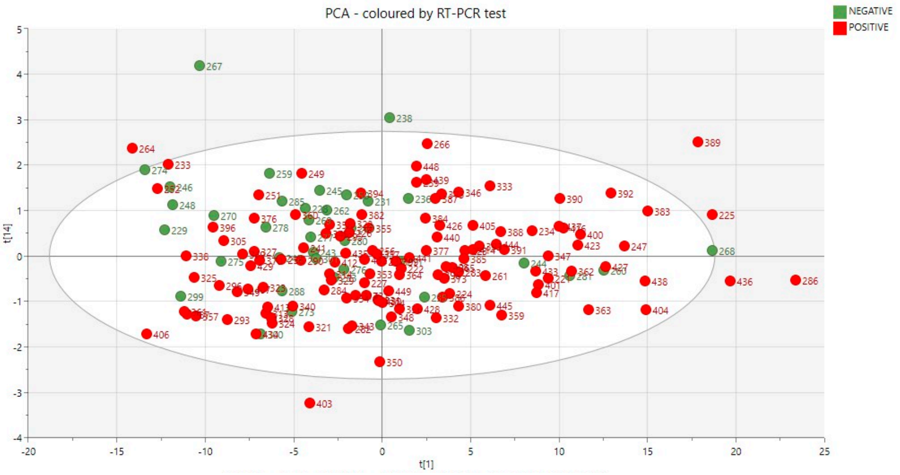

Supplementary Figure S1: Principal component analysis (PCA) of all of the data (n=164) colour coded by RT-PCR test result where red data points are positive patients (n=193) and green are patients who tested negative (n=41)

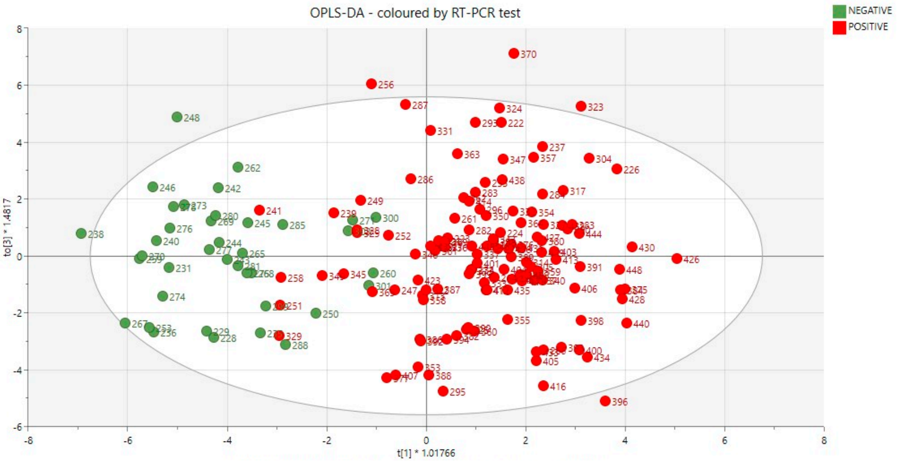

Supplementary Figure S2: Orthogonal partial least squares discriminant analysis (OPLS-DA) of all of the data (n=164) colour coded by RT-PCR test result, after reclassifying the three prior positives to positive data points, where red data points are positive patients (n=197) and green are patients who tested negative (n=37)

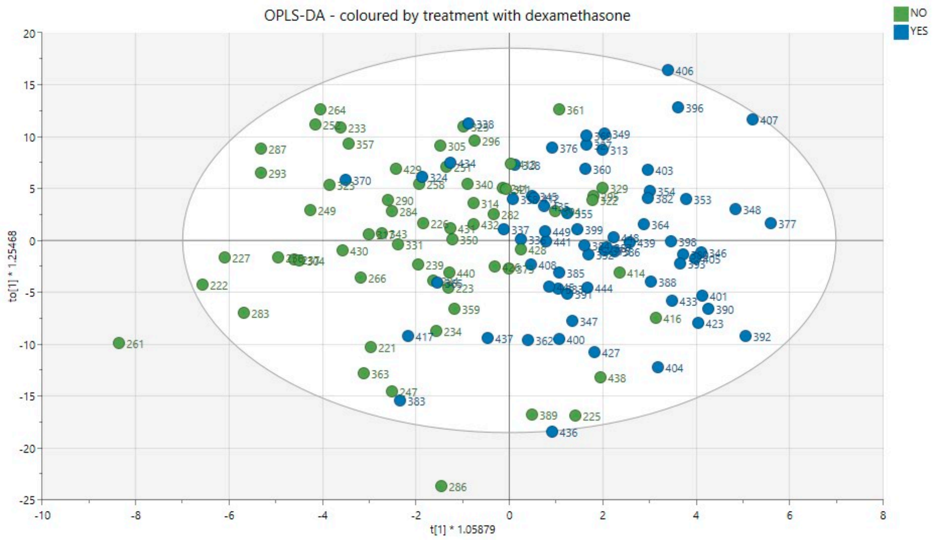

Supplementary Figure S3: Orthogonal partial least squares discriminant analysis (OPLS-DA) of the patients with a positive RT-PCR test colour coded by whether they were treated with dexamethasone (YES – blue) or not (NO – green)

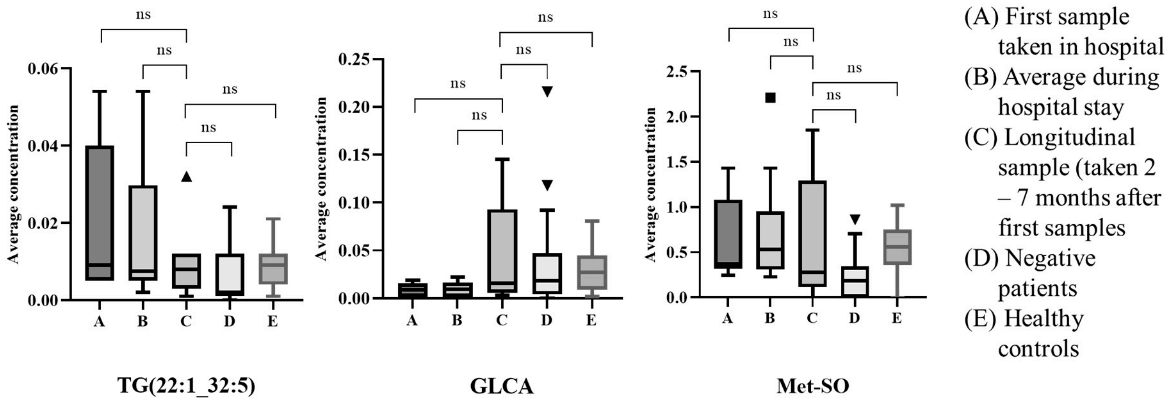

Supplementary Figure S4: Box plots to show the 3 metabolites that show no significant difference after recovery for the 7 patients with longitudinal samples where (A) First sample taken in hospital, (B) average of samples over hospital stay, (C) longitudinal samples, (D) all negative patients and (E)) Healthy controls (ns – not significant)
